# Evaluating the therapeutic effect of lenvatinib against advanced hepatocellular carcinoma by measuring blood flow changes using contrast‐enhanced ultrasound

**DOI:** 10.1002/cnr2.1471

**Published:** 2021-06-09

**Authors:** Naoki Kamachi, Masahito Nakano, Shusuke Okamura, Takashi Niizeki, Hideki Iwamoto, Shigeo Shimose, Tomotake Shirono, Yu Noda, Ryoko Kuromatsu, Hironori Koga, Takuji Torimura

**Affiliations:** ^1^ Division of Gastroenterology, Department of Medicine Kurume University School of Medicine Fukuoka Japan

**Keywords:** early stage, modified RECIST, peak intensity, renal function, time curve analysis

## Abstract

**Background:**

The antitumor effect of a drug is considered to be associated with a decrease in tumor blood flow.

**Aims:**

We investigated whether the efficacy of lenvatinib (LEN) could be accurately assessed by measuring blood flow in hepatocellular carcinoma (HCC) during early treatment stages.

**Methods and results:**

Blood flow changes and treatment results of 19 patients who underwent contrast‐enhanced ultrasound (CEUS), before and after LEN administration, in Kurume University Hospital from July 2018 to June 2020 were examined. Blood flow was evaluated after the intravenous administration of perflubutane (0.015 ml/kg). The vascular phase was photographed and used as RAW data, and time‐intensity curve analysis was used to obtain the region of interest (ROI) on the entire tumor nodule and quantify tumor blood flow. The evaluation was performed before and 1 and 4 weeks after LEN administration. Mean ± standard deviation (SD) values of the brightness of blood flow in the background liver before and 1 and 4 weeks after LEN administration were 2.84 × 10^−4^ ± 2.94 × 10^−4^, 3.07 × 10^−4^ ± 3.79 × 10^−4^, and 10.0 × 10^−4^ ± 20.8 × 10^−4^ dB, respectively. Blood flow in the background liver did not significantly decrease at 1 and 4 weeks compared with that before treatment. Mean ± SD values of the brightness of blood flow in HCC before and 1 and 4 weeks after administration were 3.49 × 10^−3^ ± 4.58 × 10^−3^, 1.16 × 10^−3^ ± 1.57 × 10^−3^, and 6.39 × 10^−3^ ± 22.8 × 10^−3^ dB, respectively. Blood flow in HCC after 1 week was significantly lower than that before administration (*p* = .0192). The therapeutic effects were significantly higher in the group with ≥50% blood flow reduction in HCC at 1 week after administration (*p* = .0038) and the group with reduced blood flow in HCC at 4 weeks after administration (*p* = .0051) than those before administration.

**Conclusion:**

Early blood flow evaluation by CEUS may be useful in predicting the therapeutic effect of LEN for unresectable advanced HCC.

## 1 INTRODUCTION

Liver cancer is the third leading cause of death worldwide and the sixth most commonly diagnosed cancer.^1^ Hepatocellular carcinoma (HCC) accounts for four‐fifth of the primary liver cancer cases.^1^ Although antiviral treatments contribute to the prevention of HCC, patients are often diagnosed in an advanced stage.^2^ With the advent of molecularly targeted therapeutic agents (MTAs), the treatment of HCC has substantially improved.^3^

In March 2018, lenvatinib (LEN), an MTA, was approved for the treatment of unresectable HCC following sorafenib and regorafenib treatment, as the first‐line treatment for patients with unresectable HCC in the United States of America, the European Union, Japan, and China, based on the results of the REFLECT trial, a global multicenter randomized phase 3 trial of LEN for HCC.^4^, ^5^, ^6^, ^7^ It has been reported that treatment with LEN results in a decrease in tumor blood flow during the early stages of administration.^4^ As the antitumor effect is induced by a decrease in tumor blood flow, early blood flow evaluation may be effective in predicting the therapeutic effect.^8^

Contrast‐enhanced ultrasound (CEUS) with perflubutane provides dynamic real‐time imaging with high spatial and temporal capabilities.^9^ Therefore, CEUS can enable to perform real‐time blood flow evaluation. In addition, as perflubutane is excreted during exhalation, it can be used even in patients with renal function impairment.

Although the therapeutic evaluation of sorafenib for HCC using CEUS has been reported, there are only a few reports on LEN.^10^, ^11^, ^12^, ^13^ We hypothesized that blood flow evaluation by CEUS is useful in predicting the therapeutic effect of LEN. In this study, we investigated whether the therapeutic effect of LEN could be accurately assessed using the minimally invasive measurement of blood flow in HCC during the early stages of treatment.

## 2 MATERIAL AND METHODS

### 2.1 Study design

This prospective study was conducted in Kurume University Hospital. The protocol conformed to the ethical guidelines of the 1975 Declaration of Helsinki and was approved by the ethics committees of Kurume University School of Medicine (18069). An opt‐out approach was employed to obtain informed consent from the patients, and personal information was protected during data collection.

### 2.2 Patients

Blood flow changes and treatment results of 19 patients, who underwent CEUS after the administration of LEN from July 2018 to June 2020 in Kurume University Hospital, were examined.

### 2.3 Inclusion and exclusion criteria

The inclusion criteria for selecting patients were as follows: patients with unresectable HCC and intrahepatic lesions, patients who could receive LEN for more than 4 weeks, patients whose blood flow could be evaluated by CEUS before and 1 and 4 weeks after administration. Patients with a history of hypersensitivity to egg or perflubutane components and those for whom perflubutane was contraindicated were excluded.

### 2.4 Method of contrast‐enhanced ultrasound

Blood flow evaluation was performed using an ultrasonic diagnostic device, Aplio 500 (Canon Medical Systems Corporation, Tochigi, Japan), after the intravenous administration of 0.015 ml/kg perflubutane. The vascular phase was photographed and used as RAW data, and the time‐curve analysis (TCA) was used to set the ROI on the entire tumor nodule and quantify the tumor blood flow. In addition, the ROI was set in the background liver to compare and evaluate the tumor blood flow and background liver blood flow. The ROI of the background liver was set in the nontumor region at the same depth as the tumor. US settings, gain, and mechanical index for each patient were set under the same conditions. The ROI of the HCC and background liver was set to avoid large blood vessels. The evaluation was performed before and 1 and 4 weeks after administration.

### 2.5 Administration of LEN

As a general rule, LEN was orally administered at a dose of 12 mg for a body weight of 60 kg or more and 8 mg for a body weight of less than 60 kg, once a day. Dose reduction was allowed according to the patient's condition. When adverse events of grade 3 or higher were observed according to the Common Terminology Criteria for Adverse Events (CTCAE) diagnostic criteria, the dose of LEN was reduced, or LEN was discontinued.^14^, ^15^, ^16^

### 2.6 Assessment of tumor response

The therapeutic effect of LEN on the tumor was evaluated using modified Response Evaluation Criteria in Solid Tumors (RECIST), based on computed tomography (CT) images taken before and at 4 weeks after LEN administration.^17^, ^18^ The results were compared and assessed with blood flow evaluation by CEUS taken before and at 1 and 4 weeks after administration.

### 2.7 Time‐intensity curve (TIC)

**FIGURE 1:**
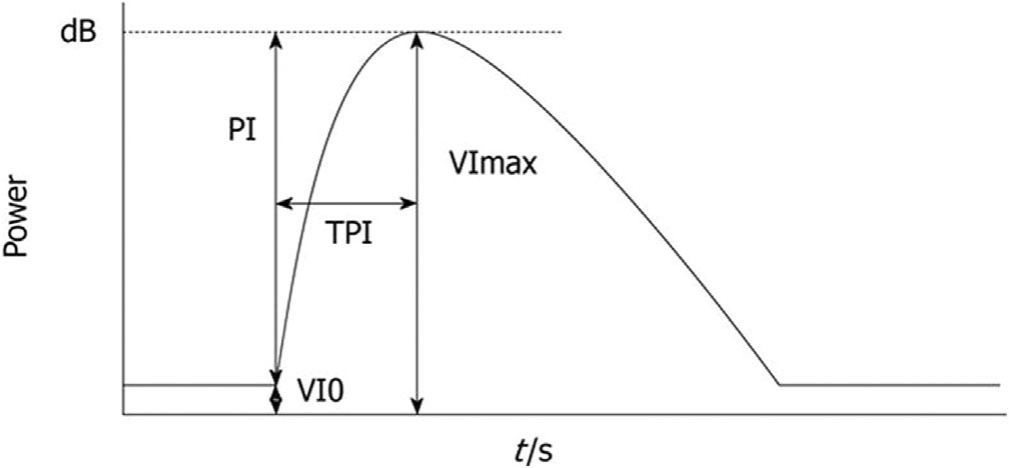
Time‐intensity curve. VI 0 is the baseline intensity of the region of interest (ROI), VI max is the maximal signal of the ROI, PI is the difference between VI max and VI 0 (VI max‐VI 0), and time to peak intensity (TPI) is the time from the onset of tumor contrast enhancement to VI max^19^

The evaluated nodules were not necessarily those with the largest tumor diameter and were within 8 cm of the body surface. Images were recorded digitally on an optical disc and analyzed off‐line. The average video intensity (VI) of the ROI in every frame was measured automatically using Image Lab (Canon Corporation, Tokyo, Japan), after the identification of the ROI that included the most markedly enhanced area of the whole tumor region. Necrotic regions, vessels, and liver parenchyma surrounding the tumor were excluded from the ROI. The corresponding TIC was plotted. The following parameters of blood flow were measured from the TIC: baseline intensity of ROI (VI 0), maximal signal of the ROI (VI max), peak intensity (PI) that showed the difference between VI max and VI 0 (VI max‐VI 0), and time to PI (TPI) ‐ time required from the onset of tumor contrast enhancement to VI max (Figure 1).^19^, ^20^ The variation in TPI was the ratio of the post‐treatment TPI to the pretreatment TPI.

### 2.8 Statistical analysis

The changes in blood flow in the region of background liver or HCC, correlation with therapeutic effects, and changes in tumor TIC values were calculated using the *t*‐test. Results with a *p* value of <.05 were considered statistically significant. JMP software (SAS Institute, Inc., Cary, NC), version 14, was used for all analyses.

## 3 RESULTS

### 3.1 Patient characteristics

Table 1 shows the characteristics of the 19 enrolled patients. The mean period until CEUS in 4 weeks was 28.9 ± 5.9 days. All patients were treated with LEN for 4 weeks or more. The mean age of patients was 75.7 ± 8.6 years at the start of LEN treatment, and 13 patients (68%) were males. The liver diseases caused by HCC were hepatitis B in 0 (0%), hepatitis C in 10 (53%), and both negative in 9 (47%) patients. The breakdown of both negative cases was as follows: NASH, 3 (16%); alcoholic, 1 (5%); and unknown cause, 5 (26%) patients. The performance status was 0 in 17 patients (89%). The mean body mass index was 24.0 ± 4.3. Fourteen patients (74%) had chronic hepatitis, six patients (32%) had a cirrhotic liver, and five patients with a cirrhotic liver (26%) had Child‐Pugh class A cirrhosis. Eight patients (42%) had albumin‐bilirubin grade 1, and 18 patients (95%) had Barcelona Clinic Liver Cancer stage B or C. The mean tumor size was 28.5 ± 15.1 mm. The mean total number of nodules was 4.7 ± 4.6. The mean levels of alpha‐fetoprotein and des‐gamma‐carboxy prothrombin were 3321 ± 13 564 ng/ml and 10 680 ± 41 022 mAU/ml, respectively. The initial doses of LEN were 4, 8, and 12 mg in 1 (5%), 12 (63%), and 6 (32%) patients, respectively. One patient who was administered 4 mg LEN, as the initial dose, was petite and elderly, and we were concerned about adverse events.

**TABLE 1:** Patient characteristics

Variable	n = 19
Age (years)	75.7 ± 8.6
Sex (male/female)	13/6
Etiology (HBV/HCV/both negative)	0/10/9
Both negative (NASH/alcoholic/unknown)	3/1/5
Performance status (0/1)	17/2
Body mass index	24.0 ± 4.3
Chronic hepatitis/cirrhotic	14/5
Child‐Pugh score (5/6)	2/3
ALBI grade (1/2)	8/11
BCLC stage (A/B/C)	1/16/2
Tumor size (mm)	28.5 ± 15.1
Total number of nodules	4.7 ± 4.6
AFP (ng/ml)	3321 ± 13 564
DCP (mAU/ml)	10 680 ± 41 022
Initial lenvatinib dose (4 mg/8 mg/12 mg)	1/12/6

Abbreviations: AFP, alpha‐fetoprotein; ALBI, albumin‐bilirubin; BCLC, Barcelona Clinic Liver Cancer; DCP, Des‐gamma‐carboxy prothrombin; HBV, hepatitis B virus; HCV, hepatitis C virus.

*Note*: Results are expressed as mean ± SD.

### 3.2 Clinical example of a good responder

**FIGURE 2:**
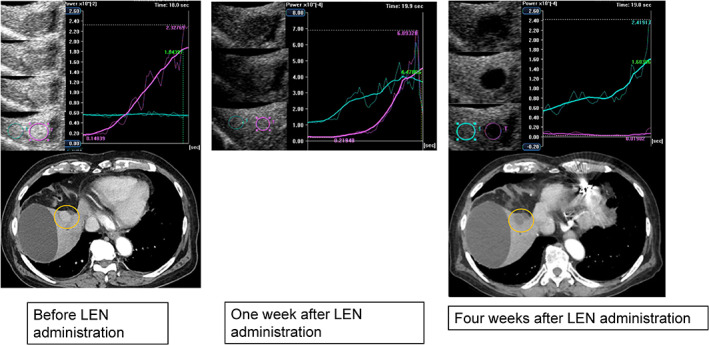
Clinical example of a good responder. An 87‐year‐old man with a history of hepatitis non‐B, non‐C virus cirrhosis underwent central hepatic bisection resection, and transcatheter arterial chemoembolization (TACE) for advanced hepatocellular carcinoma. Subsequently, he underwent radiofrequency ablation (RFA) under laparotomy for HCC recurrence. After RFA under laparotomy, the biliary cyst was complicated. HCC recurrence was observed in S5. TACE was difficult because of the presence of bile cysts. Radiation therapy was difficult near the intestine, and LEN administration (8 mg/day) was initiated. Gray‐scale ultrasonography showed a mosaic‐pattern tumor, of diameter 23 mm, in S5. This tumor was established as the target lesion. Images were taken with blood vessels and cysts near the tumor as landmarks. The left image was taken before LEN administration, the center was taken 1 week after LEN administration, and the right image was taken and TCA was performed 4 weeks after LEN administration. In each period, the CEUS, TCA, and CT were used for evaluation. TCA could measure the brightness of perflubutane. The brightness unit was automatically adjusted according to the numerical values of the HCC and background. Before administration, the peak intensity of the tumor was higher than that of the background liver. One week after LEN administration, the TCA results of the tumor and background liver were similar. Four weeks after LEN administration, it was reversed and the TCA result of the background liver was higher than that of the tumor. Four weeks after LEN treatment, a dynamic CT scan in the arterial phase showed a hypovascular lesion in S5, which was reduced. This therapeutic response was described as a partial response

Figure 2 shows images before and at 1 and 4 weeks after LEN administration. The TCA was measured by setting the ROI on the tumor and nontumor parts. Before administration, the tumor area presented a higher brightness than the non‐tumor area. One week after LEN administration, the difference in brightness between the tumor and nontumor parts decreased. Four weeks after administration, the tumor presented a lower brightness than the nontumor part. CT showed tumor enhancement before administration, but it disappeared 4 weeks later.

### 3.3 Changes in blood flow before and 1 and 4 weeks after LEN administration

**FIGURE 3:**
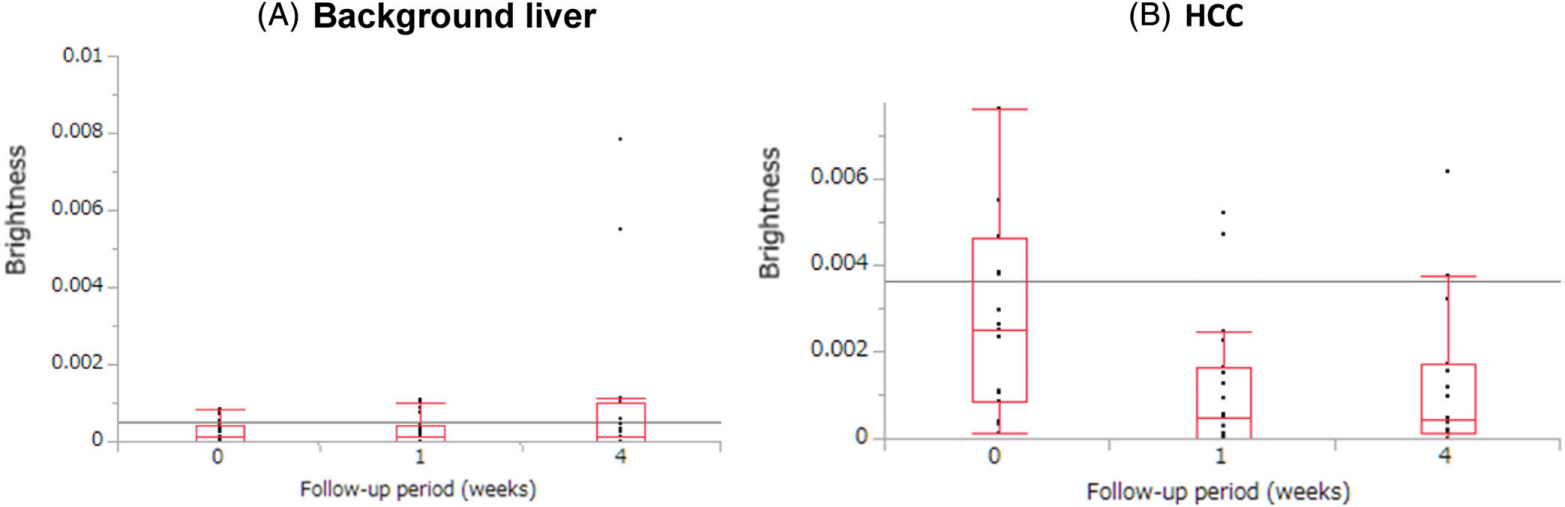
Change in blood flow before LEN administration and at 1 and 4 weeks after LEN administration (mean value ± standard deviation (SD)). (A) Region of background liver. Before: 2.84 × 10^−4^ ± 2.94 × 10^−4^, 1 week after: 3.07 × 10^−4^ ± 3.79 × 10^−4^, 4 weeks after: 10.0 × 10^−4^ ± 20.8 × 10^−4^ dB. Before versus 1 week after: *p* = .6721, before versus 4 weeks after: *p* = .5891, 1 week after versus 4 weeks after: *p* = .4221. (B) Region of hepatocellular carcinoma (HCC). Before: 3.49 × 10^−3^ ± 4.58 × 10^−3^, 1 week after: 1.16 × 10^−3^ ± 1.57 × 10^−3^, 4 weeks after: 6.39 × 10^−3^ ± 22.8 × 10^−3^ dB. Before versus 1 week after: *p* = .0076, before versus 4 weeks after: *p* = .0319, 1 week after versus 4 weeks after: *p* = .8267

Mean ± standard deviation (SD) values of the brightness of blood flow (PI) in the background liver before and 1 and 4 weeks after LEN administration were 2.84 × 10^−4^ ± 2.94 × 10^−4^, 3.07 × 10^−4^ ± 3.79 × 10^−4^, and 10.0 × 10^−4^ ± 20.8 × 10^−4^ dB, respectively (Figure 3(A)). The blood flow did not significantly decrease after 1 and 4 weeks (*p* = .6721, *p* = .5891, respectively) compared with that before administration.

Mean ± SD values of the brightness of blood flow (PI) in HCC before and at 1 and 4 weeks after LEN administration were 3.49 × 10^−3^ ± 4.58 × 10^−3^, 1.16 × 10^−3^ ± 1.57 × 10^−3^, and 6.39 × 10^−3^ ± 22.8 × 10^−3^ dB, respectively (Figure 3(B)). The blood flow after 1 week was significantly lower than that before administration (*p* = .0385). The blood flow after 1 and 4 weeks was significantly lower than that before administration (*p* = .0076, *p* = .0319, respectively).

### 3.4 Correlation between the therapeutic efficacy of LEN and changes in tumor TIC values

Table 2 shows the correlation between the therapeutic effects of LEN and changes in tumor TIC values before and 1 week after LEN administration. The mean blood flow value was 56% lower after 1 week of LEN administration. Therefore, the patients were divided into two groups as follows: the group with <50% reduction in blood flow (*n* = 6, 32%) and the group with ≥50% reduction in blood flow (*n* = 13, 68%). In the group with ≥50% reduction in blood flow, complete response (CR), partial response (PR), stable disease (SD), and progressive disease (PD) were observed in 1 (5%), 10 (53%), 2 (11%), and 0 (0%) patients, respectively, and the objective response rate (ORR) and disease control rate (DCR) were 58% and 68%, respectively. In the group with <50% reduction in blood flow, CR, PR, SD, and PD were observed in 0 (0%), 0 (0%), 3 (16%), and 3 (16%) patients, respectively, and the ORR and DCR were 0% and 20%, respectively. Based on the changes in tumor TIC values before and at 1 week after LEN administration, a significant difference in the therapeutic effects was observed (*p* = .0038). Moreover, the group with ≥50% reduction in blood flow presented significantly better ORR (*p* = .0005) and DCR (*p* = .0055) than the group with <50% reduction in blood flow.

**TABLE 2:** Correlation between therapeutic effects and changes in tumor TIC values before and at 1 week after lenvatinib administration

Therapeutic effect	≥50% reduction (*n* = 13)	<50% reduction (*n* = 6)	*p*‐value
CR	1 (5%)	0 (0%)	
PR	10 (53%)	0(0%)	
SD	2 (11%)	3 (16%)	
PD	0 (0%)	3 (16%)	.0038
Response (CR + PR)	11 (58%)	0 (0%)	.0005
Disease control (CR + PR + SD)	13 (68%)	3 (16%)	.0055

Abbreviations: CR, complete response; PD, progressive disease; PR, partial response; SD, stable disease; TIC, time‐intensity curve.

Table 3 shows the correlation between the therapeutic effect and changes in tumor TIC values before and at 4 weeks after LEN administration. The patients were divided into two groups as follows: the decreased (*n* = 14, 74%) and increased (*n* = 5, 26%) groups. In the group with a decrease, CR, PR, SD, and PD were observed in 1 (5%), 10 (53%), 3 (16%), and 0 (0%) patients, respectively, and the ORR and DCR were 58% and 74%, respectively. In the group with an increase, CR, PR, SD, and PD were observed in 0 (0%), 0 (0%), 2 (11%), and 3 (16%) patients, respectively, and the ORR and DCR were 0% and 11%, respectively. Based on the results obtained for the changes in tumor TIC values before and 4 weeks after LEN administration, a significant difference in the therapeutic effect was observed (*p* = .0051). Moreover, the decreased group performed significantly better than the increased group with respect to both ORR (*p* = .0023) and DCR (*p* = .0016).

**TABLE 3:** Correlation between therapeutic effects and changes in tumors TIC values before and at 4 weeks after lenvatinib administration

Therapeutic effect	Decrease (*n* = 14)	Increase (*n* = 5)	*p*‐value
CR	1 (5%)	0 (0%)	
PR	10 (53%)	0 (0%)	
SD	3 (16%)	2 (11%)	
PD	0 (0%)	3 (16%)	.0051
Response (CR + PR)	11 (58%)	0 (0%)	.0023
Disease control (CR + PR + SD)	14 (74%)	2 (11%)	.0016

Abbreviations: CR, complete response; PD, progressive disease; PR, partial response; SD, stable disease; TIC, time‐intensity curve.

## 4 DISCUSSION

In this study, we investigated the relationship between the time‐course evaluation of tumor blood flow and efficacy evaluation after LEN administration. In addition, the ROI was set and the brightness of perflubutane was quantified to accurately examine the relationship by evaluating blood flow in the tumor.

First, we evaluated the change in blood flow at pretreatment, after 1 week, and after 4 weeks of LEN administration in the background liver and HCC. A comparison of the changes in blood flow in the background liver revealed no significant decrease in blood flow. A comparison of the changes in blood flow to HCC showed a significant decrease in blood flow at 1 week after LEN administration compared with that before administration. Based on this result, it was assumed that the change in blood flow could be captured precisely. In contrast, blood flow in the tumor was not significantly decreased at 4 weeks after LEN administration compared with that before administration. Although the reason for this observation was unclear, it may be attributed to the reduction in LEN dose during the 4‐week period after administration owing to adverse events such as fatigue or appetite loss.

Second, we evaluated the correlation between the therapeutic effects and changes in tumor TIC values at pretreatment, after 1 week, and after 4 weeks of LEN administration. It was observed that the therapeutic effect was significantly higher when the blood flow decreased by over 50% after 1 week of LEN administration. In addition, the therapeutic effect was significantly higher when the blood flow decreased after 4 weeks of administration of LEN. These results demonstrated that the evaluation with CEUS is useful for advanced HCC treated with LEN, especially in patients with impaired renal function.

To evaluate the therapeutic effect of LEN, dynamic CT and ethoxybenzyl magnetic resonance imaging (EOB‐MRI) are generally utilized. At present, other evaluation methods, including less invasive evaluation methods, are being considered at an early stage of HCC. Dynamic CT and EOB‐MRI are invasive tests that are difficult to use in patients with impaired renal function. In contrast, as the excretion route of CEUS is exhalation, it can be used in patients with impaired renal function. CEUS allows the evaluation of the therapeutic effects of drugs with better sensitivity because it is less invasive and tumor blood flow can be evaluated in real time.

Of note, in this study, we focused only on a single target lesion. Considering multicentric carcinogenesis, which is one of the carcinogenic modes of HCC, there may be a controversy regarding the therapeutic efficacy evaluated in this study. Arizumi et al. compared dynamic CT and EOB‐MRI before and at 4–6 weeks after sorafenib treatment in 158 patients with advanced HCC, and found that intratumoral blood flow can decrease even at one nodule.^8^ They reported a significant prolongation of overall survival compared with that in the unreduced group.^8^ Therefore, the single‐nodule comparison in this study may be feasible in determining the therapeutic effect. However, it is arguable whether the evaluation of one nodule among multiple nodules on dynamic CT and EOB‐MRI and the evaluation of one nodule in one cross‐section in the US can be judged to be equivalent.

Generally, the evaluation of therapeutic effects of LEN in advanced HCC is performed by dynamic CT or EOB‐MRI, and the evaluation interval of therapeutic effect, using modified RECIST, is at least 4 weeks.^18^ With these evaluation methods, there is a risk of straining renal function, and it may not be possible to capture early blood flow changes in advanced HCC with LEN. In contrast, CEUS can be performed at any time without burdening renal function. In this study, we found that tumor blood flow significantly decreased after 1 week of LEN administration. In addition, the >50% decrease in tumor blood flow after 1 week and the decrease in tumor blood flow after 4 weeks correlated with the therapeutic effect. Therefore, the confirmation of the decrease in tumor blood flow by CEUS may be used as an index to decide whether to continue LEN treatment or not. Similarly, the confirmation of increased tumor blood flow using CEUS may be used as an index to switch LEN to secondary agents such as sorafenib, regorafenib, and ramucirumab.^21^

Our current study had some limitations. First, the size of the study cohort was relatively small. The reasons for small sample size were difficulty in evaluating tumor blood flow using CEUS (since most of the target lesions were located deep) and difficulty in receiving patient informed consent. Therefore, it is necessary to include more number of cases in future research. Second, as the blood flow to the background liver and tumor differs between patients, it was difficult to compare the absolute values. Thus, the changes in blood flow over time were evaluated for each patient. The changes in blood flow among patients over time were compared not using the absolute values but using the ratio. Third, there was no description about the “time to wash‐in” and the “time to wash‐out” in this study. Although we measured the “time to wash‐in,” no significant result was obtained; hence, we did not measure the “time to wash‐out.” We consider it is necessary to investigate a greater number of patients, including those with changes in PI, dose intensity, and tumor response.

## 5 CONCLUSIONS

The change in blood flow at week 1 and the therapeutic effect were significantly related to whether the blood flow in the tumor was reduced by ≥50% compared with that before administration. In addition, the change in blood flow at 4 weeks and the therapeutic effect were significantly related to whether the blood flow was decreased or not compared with that before LEN administration. Thus, blood flow evaluation by CEUS at an early stage of LEN treatment may be useful in determining the therapeutic effect of LEN treatment for unresectable advanced HCC.

## CONFLICT OF INTEREST

The authors have stated explicitly that there are no conflicts of interest in connection with this article.

## AUTHORS' CONTRIBUTIONS

All authors had full access to the data in the study and take responsibility for the integrity of the data and the accuracy of the data analysis. *Conceptualization*, N.K., M.N., S.O., H.K.; *Data Curation*, N.K., M.N., S.O., H.K., T.N., H.I., S.S., T.S., Y.N., R.K.; *Formal Analysis*, N.K., M.N., S.O.; *Writing ‐ Original Draft*, N.K., M.N.; *Writing ‐ Review & Editing*, N.K., M.N.; *Supervision*, R.K., H.K., T.T.

## ETHICAL STATEMENT

This prospective study was conducted in Kurume University Hospital. The protocol conformed to the ethical guidelines of the 1975 Declaration of Helsinki and was approved by the ethics committees of Kurume University School of Medicine (18069). Patients were given comprehensive information on the details of the clinical study, and they provided written informed consent prior to participation.

## Data Availability

The data that support the findings of this study are available from the corresponding author upon reasonable request.
